# Assisted Tip Sonication Approach for Graphene Synthesis in Aqueous Dispersion

**DOI:** 10.3390/biomedicines6020063

**Published:** 2018-05-28

**Authors:** Ahmed F. Ghanem, Mona H. Abdel Rehim

**Affiliations:** Packaging Materials Department, National Research Centre, Elbehoth Street 33, Dokki, Cairo 12622, Egypt; info@nrc.sci.eg

**Keywords:** graphene, chemical method, liquid dispersion approach, electrical conductivity

## Abstract

Graphene (G) is a newcomer material that holds promising properties for many applications. The production of high quality G with a good yield is a long-standing goal for many researchers. This work emphasizes synthesis of dispersed graphene nanoplatelets (DGP) through aqueous dispersion technique in surfactant/water solution with the aid of tip sonication. A chemical method was also used to prepare graphene oxide (GO) and reduced graphene oxide (RGO) for comparison. Elemental analysis revealed the C:O ratio to be 12:1 for DGP but much lower for other graphene structures. Optical characterization of DGP, GO and RGO with UV and Raman spectroscopy confirmed the ideal structure of DGP. Moreover, X-ray diffraction (XRD) revealed the amorphous structure of DGP. Transmission electron microscope (TEM) imaging showed that DGP was composed of a few flat layers, unlike the wrinkled and partially bent multilayered G. Topological study of the DGP surface with scanning electron microscope (SEM) depicted its rough surface with (r_a_) value of 35 nm, as revealed using an atomic force microscope (AFM). Electrochemical measurements confirmed the higher conductivity of DGP over graphene prepared by chemical method due to lack of structural defects. Its perfect structure facilitates the mobility of charge carriers that makes it preferable in optoelectronic applications.

## 1. Introduction

Graphene (G) is a two-dimensional allotrope of carbon of great research interest since its discovery in 2004 [1]. The nanosheet is formed from one atom thick hexagonally arranged carbon atoms. This structure gives G its unusual thermal and electronic properties [2,3] along with extreme mechanical stiffness and large surface area that is theoretically predicted to be >2500 m^2^ g^−1^ [4,5,6]. Due to these remarkable properties, G has found applications in different areas such as sensors [7], gas and energy storage [8,9,10,11], optoelectronics [12], polymer composites and catalysis [13,14]. Other explored technological applications such as solar cells, light emitting devices, photodetectors and touch screens have been reported [15,16,17,18,19]. Synthesis of high quality G but in limited quantities can be performed using chemical vapor deposition (CVD) [20] or building the nanosheets from its molecular building units [21]. However, production of defect-free G by ball milling or micromechanical cleavage of ordered graphite [22,23] has been described. Moreover, a chemical method has been reported in which preparation of high yield G can be carried out via a two-step technique. First, graphite is oxidized with a suitable oxidizing agent to get graphene oxide (GO). Second, the formed GO is reduced to obtain G nanosheets [24,25]. Although the advantage of this method is the production of large amounts of G, the formed nanosheets suffer from the presence of defects in the form of carboxylic and carbonyl groups, localized atoms defects and non-reduced functional groups. The need for large amounts of high quality G suitable for advanced technological applications has forced researchers to explore other preparation techniques such as liquid-phase exfoliation of graphite, a method that has been reviewed [26,27]. This fabrication technique is based on sonication of graphite flakes in the presence of a suitable surfactant to prevent re-aggregation of the formed nanosheets [28,29,30]. Exfoliation of graphite in organic solvents followed by ultra-sonication in the absence of surfactant was also described [31,32,33,34,35]. Solvents ideal for this process can minimize the interfacial tension between liquid and G through minimizing the area of the surfaces in contact [36].

Unfortunately, the best solvents for successful dispersion of G are *N*-methyl-2-pyrrolidone (NMP), *N*,*N*-dimethylformamide (DMF), and ortho-dichlorobenzene (o-DCB), all of which have high boiling points and are irritating or highly toxic [27]. Water is an environmentally friendly solvent with moderate boiling point that can replace the organic solvents; however, due to the hydrophobic nature of G, a surfactant must be used during exfoliation to keep G nanosheets suspended in water [37,38,39]. Different factors affect exfoliation of graphite during G synthesis such as graphite/surfactant concentration and sonication time. Recent work has been done to obtain high quality G nanosheets in large quantity utilizing a sonication approach. For example, Noroozi et al. prepared dispersed graphene by ultra-sonication of graphite in a solution mixture of hand soap and Polyvinylpyrolidone [40]. However, the good dispersion of the obtained G, the prepared films based on G still suffered from high resistance and lower conductivity. Moreover, the preparation steps are lengthy. Some reports have been published to utilize sodium dodecylbenzenesulfonate (SDS) for better dispersion of graphene in an aqueous solution. Takayanagi et al. could resolve graphene and chemically oxidized graphene using Micellar Electrokinetic Chromatography with sodium dodecylbenzenesulfonate as a micelle matrix. The results confirmed successful dispersion of graphene nanoplatelets and a consecutive broad signal was obtained with 20 mmol dm^−3^ an aqueous SDS solution [41]. Yeari et al. successfully synthesized reduced graphene oxide with SDS as an exfoliator and stabilizer at the same time via a simple ultrasonication method [42]. Even though good results were obtained in both water and organic solvents, this process consumed much time in terms of preparation of graphene oxide followed by reduction with hydrazine in presence of SDS. It is considered non-environmentally friendly due to the use of harmful reagents. In addition, the yield is quite poor and non-preferable for large scale synthesis. It could be claimed that the most common technique, which involves the oxidation and subsequent reduction of graphene oxide in presence of SDS to obtain highly dispersed graphene nanoplatelets, suffers from significant disadvantages: first, the long steps of preparation and consuming hazardous chemicals; and, second, the oxidation followed by reduction process results in the formation of structural defects which virtually alter the electronic structure of graphene and hence render its electrical conductivity. These defects are impossible to remove completely even after annealing or chemical treatments. Thus, the most promising technique to give high quality dispersed graphene at reasonably good yield is the direct exfoliation of graphite in the liquid phase such as water. An interesting study was performed to exfoliate graphite in SDS solution with the aid of ultrasound [43]. It is the first attempt for aqueous surfactant based exfoliation with sodium dodecylbenzenesulfonate. Even though transmission electron microscopy showed mixture of mono- and multilayer graphene sheets, small graphitic flakes were observed that might be due to short sonication time. A recent article has described the preparation of liquid phase exfoliated graphene with SDS under mild conditions [44]. Nevertheless, the yield of the obtained graphene is still a challenge. A theoretical study by Yoon et al. revealed that the exfoliation energies vary with the size of intercalant and the interaction with graphite [45]. Moreover, intercalation of electronegative or electropositive intercalant increases exfoliation energy due to additional binding forces through charge transfer from intercalant and host graphite.

Several surface stabilizers have been examined for exfoliation of graphite such as polycyclic aromatic hydrocarbons [46], sodium cholate (SC) [37,47], sodium dodecyl sulfate [48] and even conventional polymers [49,50,51]. 

We examined exfoliation of graphite in water with the aid of sodium dodecyl benzene sulfonate (SDS) to prepare DGP using a tip sonication technique. A few layers of defect free G (>5) were successfully prepared, as confirmed with high resolution transmission electron Microscope (HRTEM). In addition, Raman spectroscopy and elemental analysis confirmed the low concentration of oxide in the prepared nanosheets. AFM emphasized the high surface roughness and large topographic height. Thus, this DGP can be used to prepare conductive thin films on glass substrate to replace Indium tin oxide (ITO) as conductive oxide for photoelectric applications. An extensive electrochemical study of the DGP and its analog prepared with the Hummer method revealed the superior electrical properties of the G synthesized in aqueous solution.

## 2. Experimental Section

### 2.1. Materials

Sodium dodecylbenzenesulfonate (SDS) and sulfuric acid (99.9%) were purchased from Sigma Aldrich (Germany). Graphite (GPH) powder (99.9%) was provided by Fisher Scientific UK. Potassium permanganate (>99%) and hydrogen peroxide (30%) were bought from Bio Basic Canada Inc. and Carl Roth GmbH, respectively. Sodium nitrate (99.99%) and hydrazine hydrate (99%) were supplied by Sd Fine-CHEM limited (India). All chemical substances in this work are commercially available and were used as received.

### 2.2. Techniques

Scanning electron microscopy (SEM) measurements were obtained using a Zeiss SEM Ultra 60 field emission scanning electron microscope (FESEM), Königsallee 9-2137081 Göttingen (Germany). Elemental analysis was obtained from Vario El-Elementar, IRMS-North (Germany). The spectra of the prepared samples were collected at laser wavelength 532 nm and laser power 0.10 MW at 25 °C using Raman Spectroscopy, Bruker Optics, Rudolf-Plank-Str. 2776275 Ettlingen (Germany). AFM images were captured on Dimension 3100 (Veeco Digital Atomic Force Microscope by Bruker Optics). Samples for AFM imaging were prepared by spin coating ten layers of G colloidal suspension (0.5 wt % in dimethylforamide) on clean glass slides (2 × 2 cm^2^) at 2000 rpm for 80 s. The surface morphology of the prepared G sheets was studied using a transmission electron microscope, (JEM-1230; JEOL Ltd., Tokyo, Japan), under acceleration voltage 80 kV. X-ray diffraction (XRD) patterns of the produced solids were determined using a Bruker diffractometer (D 8 advance target). A CuK_α_ radiation source with secondly mono-chromator (λ = 1.5405 Å) at 40 kV and 40 mA was used. The scanning rate was 0.2 min^−1^ for phase identification and line broadening profile analysis, respectively. The optical microscope was manufactured by Olympus CH2 Optical Microscope, (Japan) and mounted with an Olympus SC100 camera. Electrochemical investigations including cyclic voltammetry and electrochemical impedance were carried out on Biologic Electrochemical Workstation (4. rue de Vaucanson; Seyssinet-Pariset 38170, France). A conventional glass cell containing the electrolyte solution with 2 mL of 5 mM [Fe(CN)_6_]^−3/4^ solution in 0.1 M KCl over a potential window of 1.0 V to −1.0 V at a scan rate of 100 mV/s was used for electrochemical experiments in the three-electrode system. Modified glassy carbon electrode, Pt wire, and Ag/AgCl served as the working, auxiliary, and reference electrodes, respectively. Typically, 1 mg of solid material was dispersed in 1 mL double distilled water and then 10 µL of Nafion solution was added. The working electrode was prepared by adding a 10 µL droplet of the prepared solution on the surface of the glassy carbon electrode and left to dry under vacuum for 30 min. Then, it was installed in the electrochemical cell. The electrical conductivities of the obtained graphene platelets were calculated from cyclic voltammetry in terms of µF.s/V units according to the following steps:

Measure capacitive current of oxidation and reduction process at which the area has no any peaks at different scan rates.

I (capacitive current) = (I **_oxi_** − I **_Red_**) × Scan Rate/2 (1)

The electrical conductivity is the average of the capacitive currents. Electrical conductivities at the bulk solid were recorded with a DC power supply (M30-TP305E), Mainland (China). The sample powder was compressed well between two copper plates separated by a round hollow Teflon cylinder, and then connected to the electrical cell. The values were calculated from Equation (2):

Conductivity = (L/A) ×1/R(2)

where L is thickness of the compressed sample, A is cross-sectional area and R is the resistivity released by the device.

### 2.3. Synthesis of Reduced Graphene Oxide (RGO)

The production of reduced graphene nanosheets via Hummer method involved two subsequent steps, oxidation of graphite followed by reduction of graphene oxide. The typical procedure was to stir 15 g of graphite powder in solution of 7.5 g sodium nitrate dissolved in 230 mL of concentrated sulfuric acid, and the temperature was decreased to 0 °C. Then, 45 g of potassium permanganate was slowly added to the mixture and the temperature was allowed to rise to the ambient value. The mixture was left under vigorous stirring for 3 h. Once the reaction was completed, 700 mL of distilled water was added and the temperature was kept at 98 °C. After 20 min of stirring, 2100 mL of hot distilled water was added. Then, 150 mL of hydrogen peroxide was added slowly to the previous solution to avoid effervescence. Finally, the graphite oxide suspension was sonicated for 30 min and then filtered out. The produced GO solid was washed several times with hot distilled water until pH ~7. The resultant brown paste was then collected and dried overnight at 60 °C under vacuum. To prepare reduced graphene oxide, 100 µL of hydrazine hydrate (NH_2_NH_2_) was added to GO solution (1 wt. %) and heated in the domestic microwave (1000 W for 3 min) to perform the reduction process. Finally, the formed graphene flakes were separated and washed well then dried at 80 °C overnight.

### 2.4. Synthesis of Dispersed Graphene Nanoplatelets (DGP)

**Scheme 1:**
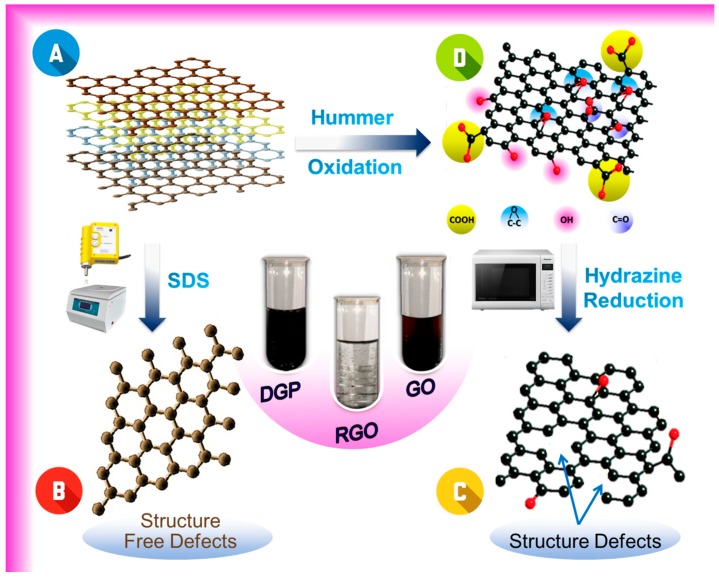
Graphical representation of graphene synthesis prepared from the Hummer method and dispersion technique using sodium dodecylbenzenesulfonate (SDS): (**A**) graphite structure; (**B**) dispersed graphene nanoplatelets; (**C**) reduced graphene oxide; and (**D**) graphene oxide.

Consuming the same precursor, dispersed graphene nanoplatelets was obtained in water via solvent dispersion method, Scheme 1. Graphite layers were exfoliated with sonication using Tip Ultra-sonicator (100 W). SDS (0.25 g) was dissolved in 150 mL deionized water and then 0.5 g of graphite was added. The graphite solution was sonicated for 12 h in an ice bath and then the suspension solution was centrifuged at 686× *g* for 30 min to remove the large particles. The precipitate was discarded and the supernatant was re-centrifuged for 90 min at 12,600× *g*. The obtained DGP was washed well several times to get rid of the surfactant. Finally, the product was dried at 60 °C under vacuum for further investigations.

## 3. Results and Discussion

Synthesis of DGP was carried out using SDS as dispersing agent because SDS as an ionic surfactant can decrease the surface energy of formed nanosheets. The concentration of graphite: surfactant was 2:1; more precisely, 3.3 mg/mL of graphite was used. Because the concentration of SDS is above its critical micelle concentration, which is 0.7 [52], a longer sonication time was used. A study for the optimum dispersion conditions revealed 0.5 mg/mL of SDS could also yield a stable aqueous dispersion [43]. However, successful exfoliation depends on other factors such as solvent and sonication time [27]. For comparison, RGO and GO prepared with chemical method were obtained as shown in Scheme 1.

Generally, G is a hydrophobic material that cannot be dispersed in water without the aid of surfactant. On the other hand, GO has hydrophilic character due to presence of carboxylic, hydroxyl, epoxy and carbonyl groups on its surface [53]. These topological functional groups are responsible for its poor electrical properties. Reduction of GO using a suitable reducing agent yields RGO [54,55,56,57]. These multistep preparation processes make production of graphene exhausting and environmentally unfriendly due to extensive use of chemicals in the oxidation and reduction steps. 

### 3.1. Elemental Analysis

Elemental analysis was performed to determine the amount of carbon and other elements on the produced graphene structures. Table 1 lists the measured values for RGO and DGP. It is noted that G obtained from dispersion method contained a higher ratio of C and a small amount of other elements such as S and O. Despite several washings, a small amount of SDS is attached to the DGP surface, which explains the presence of sulfur in the DGP sample. It could be a physical bonding was established due to π–π electrostatic interaction between the planer π-conjugated surfaces. Nevertheless, the small amount of S (0.335%) recorded for DGP compared to the amount found for RGO (2.2%) confirms the superiority of this method. Moreover, the RGO sample showed not only high sulfur content, in the form of sulfate groups due to using of sulfuric acid in the preparation step, but also high amounts of N and O. It is believed that the GO surface contains different carbonyl compounds such as anhydrides and quinones. On reduction of GO with hydrazine, hydrazides and hydrazones are formed, and only hydrazone formation can lead to removal of oxygen [58]. Moreover, ring opening reactions of epoxide groups on the GO surface and hydrazine are also possible, yielding hydrazine alcohols, but oxygen will not be removed [59]. This would explain the presence of a high ratio of oxygen in the RGO sample compared to DGP [60,61]. Calculating the C/O ratio for the RGO and DGP samples gave 2.6:1 and 12:1, respectively, which confirm the lack of oxygen-containing compounds on the surface of DGP. In addition, the small amount of N present in the DGP sample might be due to the use of dimethylforamide as a solvent to prepare thin films required for the analysis. 

### 3.2. Raman Spectra

**Figure 1:**
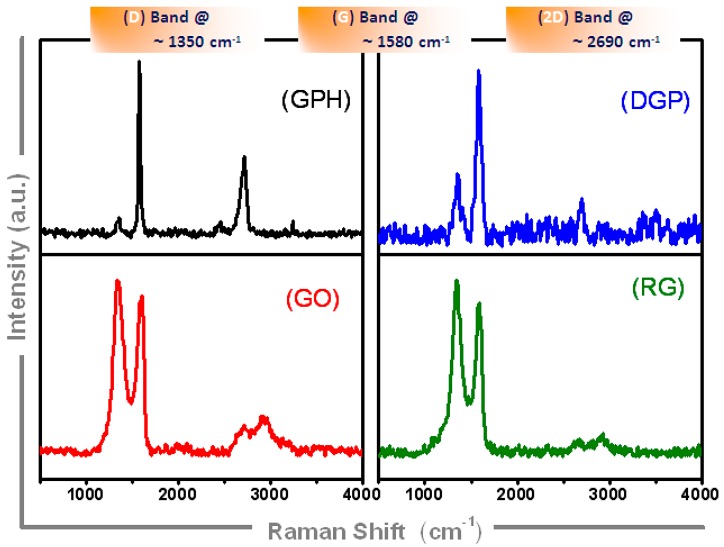
Raman spectra of graphite (GPH), dispersed graphene platelets (DGP), graphene oxide (GO) and reduced graphene oxide (RG).

The quality of exfoliated graphene prepared by either chemical or aqueous dispersion methods was characterized with Raman spectroscopy along with graphite (GPH) and GO for comparison (Figure 1). In general, the photon energy shift caused by laser excitation at 532 nm created significant peaks in the Raman spectrum. All the spectra are dominated by a main band at around 1580 cm^−1^. This peak is characteristic for GPH and G and is called the G-band. This band arises due to the first-order in-plane vibrational mode of Raman. Two other peaks are also observed due to a higher order process involving more phonons: 2D (2690 cm^−1^) and D (1350 cm^−1^) [62]. For DGP, the former band is narrower and of lower intensity than for GO or RGO obtained by the Hummer method. This may be attributed to a decrease in the degree of disorder and defects in the graphitic structure during exfoliation with SDS [43,63]. The aforementioned 2D band is similar to the graphite band while the intensity is less than the G-band. This may reveal that the produced graphene flakes are relatively thick and of more than five graphene layers. Unlike DGP and GPH, the spectra of GO and RGO showed broadening in the Raman characteristic bands. This might be attributed to the structural defects in the obtained structure. Furthermore, the intensity ratio of the D-band to the G-band indicates the quality of the produced graphitic structures because it approaches zero for highly ordered pyrolitic graphite [64,65]. The relative intensity of D- and G-bands (I_D_ and I_G_) is an estimation of graphene disorder, and the Raman spectrum of the DGP exhibits a weak disorder induced by lower D-band intensity with the I_D_/I_G_ ratio of ~0.39, as compared with 1.17 and 1.08 for GO and RG, respectively. In addition, this value mostly resembles graphite, which is the highly ordered structure of the carbon materials. D-to-G intensity ratio of graphite is ~0.2. In addition, I_D_/I_G_ ratio for our graphene is a reasonable value as compared with the state of the art. Nawaz et al. could produce graphene with sodium cholate in water [66]. The I_D_/I_G_ ratio was 0.317 after 96 h sonication time. The results confirmed that there is an increase in I_D_/I_G_ value by increasing sonication time suggesting a decrease in flake size. Several defects in graphene flakes were created by cutting the graphite and forming new edges. Khan et al. demonstrated preparation of graphene dispersions in *N*-methyl-pyrrolidone for long sonication times [67]. Raman results showed I_D_/I_G_ value increases gradually up to ~0.5 after 200 h sonication time. Lotya et al. presented production of graphene stabilized in water [37]. The I_D_/I_G_ ratio was around 0.57. This value shows that significant quantities of the graphite flakes have not been cut dramatically by prolonged sonication. Sahoo et al. synthesized graphene through exfoliation of graphite in ortho-dichloro benzene by sonication [29]. I_D_/I_G_ value of the obtained graphene was 0.015 after 4 h sonication. Obviously, a significant reduction in the degree of disorder and defect positions was observed upon exfoliation of graphite with SDS. This emphasizes the high quality of our exfoliated graphene. 

### 3.3. Infrared and Ultra-violet Spectroscopy

**Figure 2:**
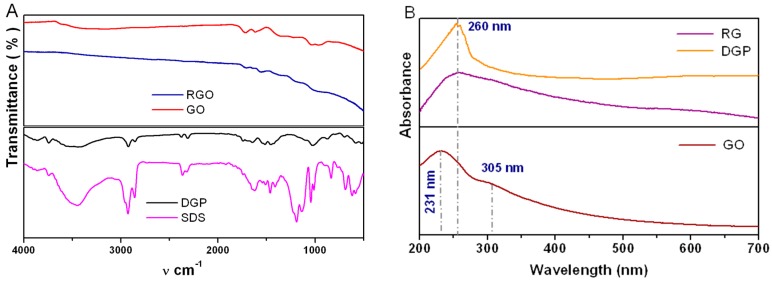
(**A**) FTIR spectra of sodium dodecylbenzenesulfonate (SDS), dispersed graphene nanoplatelets (DGP), reduced graphene oxide (RGO) and graphene oxide (GO); and (**B**) UV spectra of DGP, RG and GO.

Figure 2A shows the FTIR spectra of the different exfoliated graphene structures compared with the surfactant. The SDS spectrum shows two strong bands at 2980 cm^−1^ from the starching vibration of the CH alkyl chain, and the bands at 1220 cm^−1^ and 1090 cm^−1^ can be assigned to the symmetric and asymmetric vibrations of the sulfate groups [68]. These characteristic bands of SDS were almost absent in the spectrum of DGP. This confirms that most of the SDS molecules were eliminated by washing several times. From this result, it is clear that the interaction between DGP and SDS is a physical interaction in the form of an van der Waals forces because there is no shift in the peaks. On the other hand, the GO spectrum shows significant bands at 3360, 1730, 1610, 1340, 1040 and 1230 cm^−1^, corresponding to the stretching vibrations of the -OH, C=O, C=C, C-OH, C-O and epoxide groups, respectively [55,69]. Some bands were absent from the spectrum of the RG due to reduction by hydrazine hydrate. The others (e.g., C=O) have quite lower intensities, which confirm their presence in small amounts. As a net result, these findings are harmonious with the elemental analysis measurements.

The optical absorption spectra of GO, RG and DGP samples are shown in Figure 2B. There was an absorption band at 260 nm due to π–π* transition of the extended conjugate system of graphene for RGO and DGP [68]. However, the graphene electronic configuration was kept in the graphene platelets exfoliated with SDS. The characteristic shoulder of graphene oxide at 305 nm, attributed to n–π* transitions of C=O bonds [70], is absent in the spectra of RGO and DGP, and the GO absorption peak at 230 nm red shifts to 260 nm in the RGO. It could be claimed that the electronic conjugation within the graphene sheets is restored because of reduction with hydrazine hydrate.

### 3.4. X-ray Diffraction

**Figure 3:**
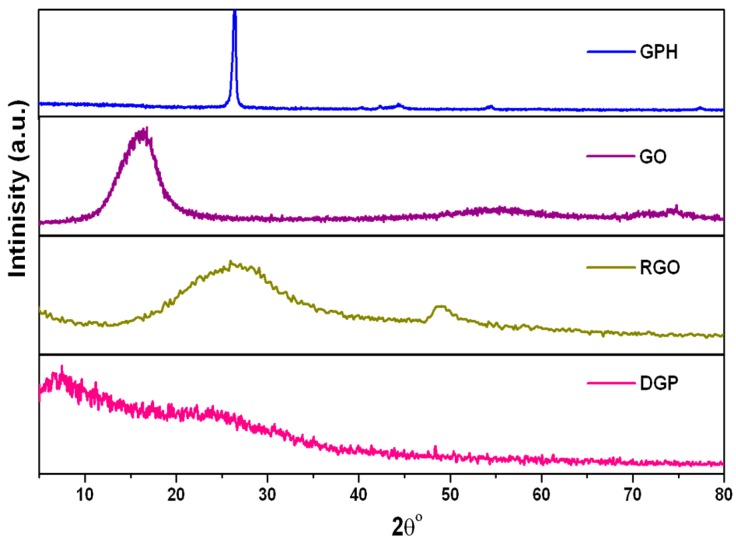
X-ray diffraction spectra of graphite (GPH), dispersed graphene platelets (DGP), graphene oxide (GO) and reduced graphene oxide (RGO).

Investigation of the prepared samples with XRD revealed the amorphous-like structure of the DGP sample (Figure 3), which confirms complete exfoliation and distortion of the graphite crystal structure. On the other hand, the XRD pattern of the GO was characterized by a peak at 2θ = 13° with a larger d-spacing resulting from the insertion of epoxy and carbonyl groups between the carbon sheets and the carboxyl and hydroxyl groups along the edges of the graphene sheet due to chemical oxidation process [71,72]. Ultimately, after reduction of GO with hydrazine hydrate, the RGO nanosheets showed plainly the disappearance of the graphene oxide peak without the re-formation of the characteristic graphite peak at 2θ = 26° corresponding to (200) reflections. This emphasizes the deoxygenation of GO sheets and the restoration of the sp^2^ carbon sites in the formed RGO.

### 3.5. Transmission Electron Microscope (TEM)

**Figure 4:**
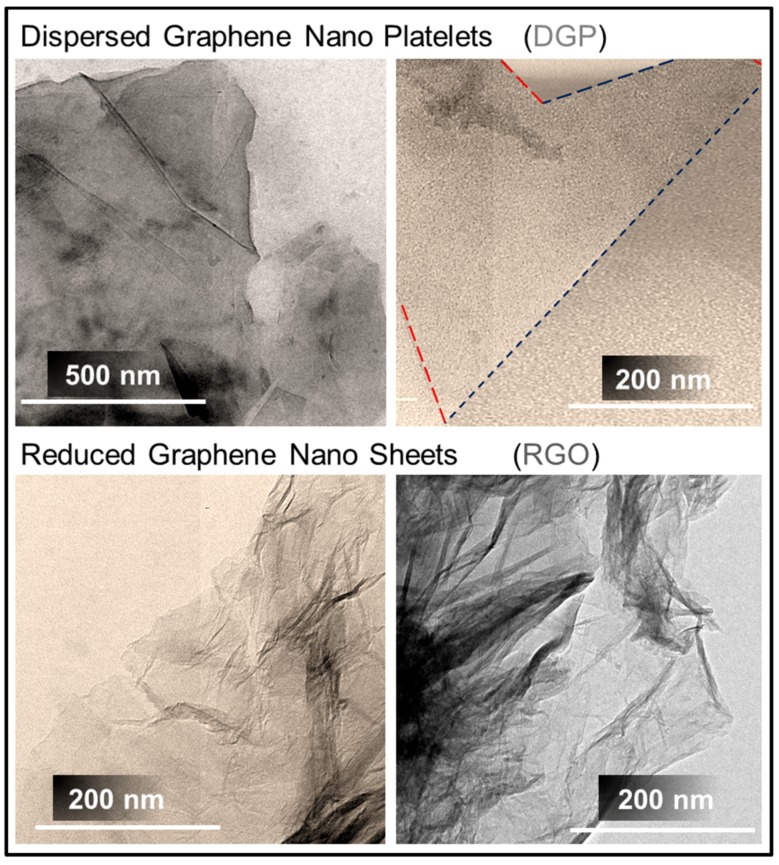
High resolution transmission electron microscope images of graphene nanosheets obtained with the aqueous phase dispersion technique and the Hummer method.

TEM micrographs of DGP confirmed successful exfoliation of the graphite using SDS (Figure 4). A mixture of mono- and multilayer graphene nanosheets was obtained (the blue and red lines indicate the edges of the monolayer). In addition, there are some black spots observed over the DGP which might be attributed to the residues of the surfactant, SDS, as it was further evidenced with elemental analysis. Moreover, the graphene sheet is shown to be relatively large-size and it has lateral dimensions of over 1 µm. On the other hand, TEM images of the RGO showed wrinkled and partially bent transparent multilayer sheet-like structure. The lateral dimension of few micrometers in length of the folded sheets is in accordance with that reported previously [68]. The morphology study depicted the better sheet quality of DGP over that of RGO.

### 3.6. Scanning Electron Microscope (SEM)

**Figure 5:**
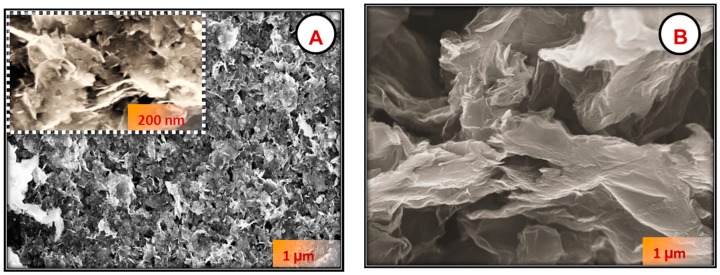
Scanning electron microscope micrographs of: (**A**) dispersed graphene nanoplatelets (inset represents higher magnification (bar = 200 nm)); and (**B**) reduced graphene oxide nanosheets.

SEM images of DGP revealed quite broken graphene sheets of rough surface (Figure 5). This may be attributed to the strong tip sonication for the long time during the preparation step. On the other hand, the surface morphology of RGO showed a crimpy, wavy and fluffy tissue-like structure that confirmed the exfoliation of compact graphite oxide structure in the reduction step with hydrazine monohydrate.

### 3.7. Atomic Force Microscope (AFM)

**Figure 6:**
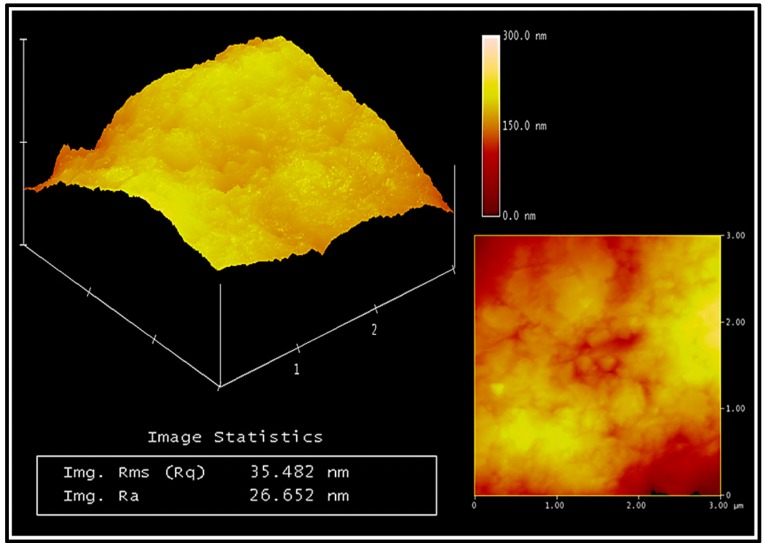
Atomic force microscope images of the dispersed graphene nanoplatelets monolayer on a glass substrate.

Once graphene nanosheets were fully characterized, there was a need to study their morphology as a film on a glass substrate and their electrochemical properties as an electrode. DGP was dispersed in dimethylforamide and spread on a clean glass substrate with a spin coating technique. The one-layer image was examined with AFM for deeper topographic view (Figure 6). The images confirmed the high surface roughness of the thin film. The mean value of surface roughness was 35 nm. It is assumed that the thickness of graphene was a little bit large and hence the topographic height was ~150 nm. These results are compatible with the Raman and TEM data. It is believed that this type of rough surface is advantageous for photoelectric devices such as solar cells.

### 3.8. Electrochemical Measurements

#### 3.8.1. Cyclic Voltammetry

**Figure 7:**
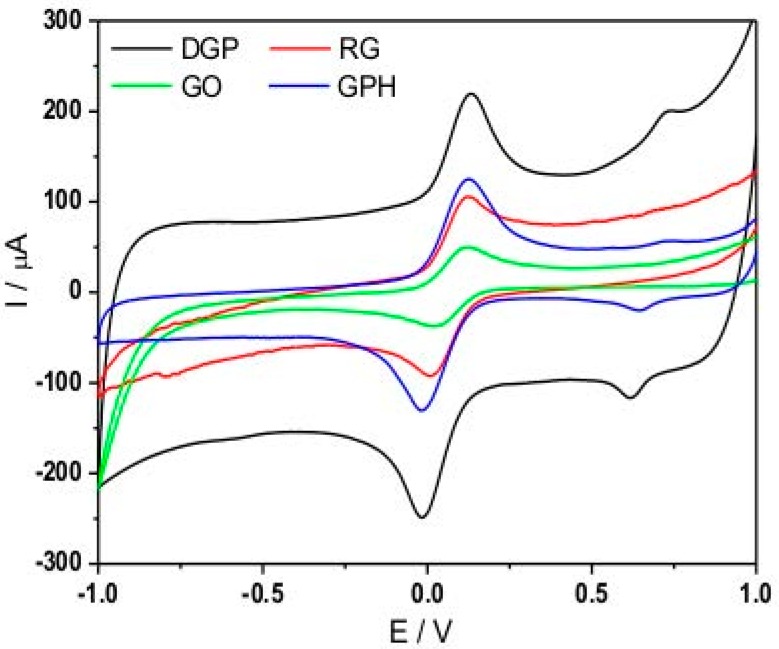
Cyclic voltammograms of graphite (GPH), dispersed graphene nanoplatelets (DGP), graphene oxide (GO), reduced graphene oxide (RGO).

Cyclic voltammetry (CV) was used to investigate the electrochemical properties of DGP compared with other graphene derivatives using drop casting technique onto a glassy carbon electrode. Figure 7 depicts the cyclic voltammograms using [Fe(CN)_6_]^−3/4^ solution as an electrolyte. One can observe oxidation peak at 0.0 V and reduction peak at 0.13 V of the DGP. Other modified electrodes showed oxidation peak at 0.01 V and reduction peak at 0.14 V (nearly at the same position as DGP). However, a significant difference in the current values was noticed exhibiting the highest current in DGP. This may be attributed to the perfect structure of DGP with absent of defective sites that hinder the mobility of the charge carriers. As aforementioned, TEM images confirmed the presence of monolayers and multilayers graphene sheets which could improve the electrical conductivity. Hence, the mobility of the electrons along the nanosheets is facilitated and led to increasing in the current. Moreover, the physical interaction between DGP and SDS in the form van der Waal forces did not change the planer structure of the nanosheet. Otherwise, decay in the current would be observed as in the case of RGO and GO. Furthermore, oxidation followed by reduction with hydrazine hydrate usually generates structural defects in the form of carbonyl, ether among others. As discussed before, the reaction of hydrazine with epoxide groups yields nitrogen containing rings [58] that hinder the electrons mobility along the sheet and leads to a decline in the current. On the other hand, CV tool was used to determine the electrical conductivity in terms of µF.s/V. The modified electrode of DGP showed good electrical conductivity compared with the other electrodes. The electrical conductivities of DGP and GPH modified electrodes were 144.84 µF.s/V and 84.9 µF.s/V, respectively. 

#### 3.8.2. Concentration/Conductivity Relationship

**Figure 8:**
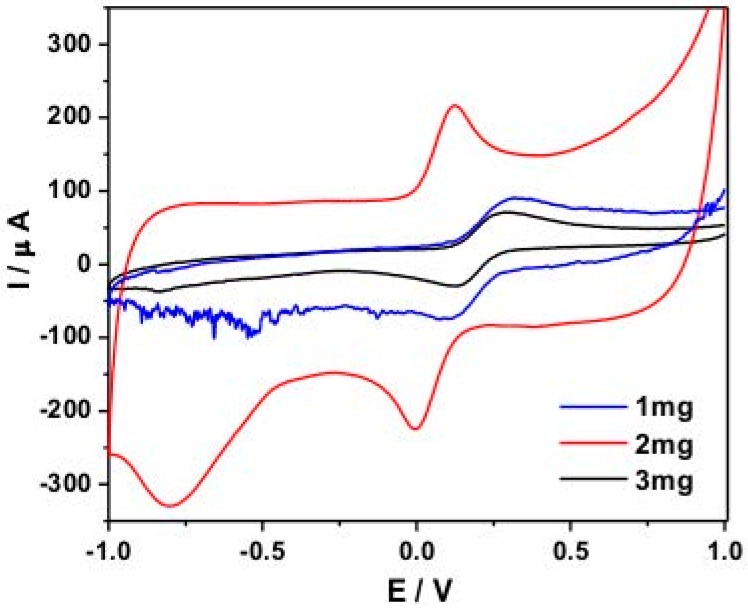
Cyclic voltammetry of DGP modified electrodes of different concentrations.

To select the best electrode, the effect of concentration was studied correlated with electrical conductivity using 1, 2 and 3 mg *wt.*/*wt.* of DGP sample within the modified electrode. From the cyclic voltammetry, the values of electrical conductivity were 61, 144 and 48 µF·s/V for 1, 2 and 3 mg *wt.*/*wt.*, respectively. Thus, the optimum concentration that exhibited the highest electrical conductivity is 2 mg (Figure 8).

### 3.9. Electrical Conductivity Measurements

Table 2 lists the electrical conductivities values in the bulk state for the graphene structures obtained. As shown, the DGP has the highest electrical conductivity compared with other samples. These results were synergetic with the electrochemical measurements and confirmed the perfect structure of DGP over that prepared with Hummer method.

## 4. Conclusions

As the production of the high quality G in good yield is the goal of many researchers, in this context, graphene nanosheets were prepared in aqueous medium using SDS as a surfactant. Characterization of the DGP obtained with elemental analysis and Raman spectroscopy confirmed the high carbon to oxygen ratio and no defects in the form of hydroxyl or carboxyl groups were detected. Successful removal of the surfactant via washing several times was confirmed by FTIR and UV spectra. TEM images revealed mono- and multilayers graphene Nano platelets obtained in aqueous dispersion. Topological investigations with AFM showed the surface roughness of the films deposited on glass substrate, which equals 35 nm. This rough surface is ideal for the solar cells’ electrodes because the incident rays would not suffer any dissipation, which in turn should increase the efficiency of the solar cell. Studying of electrochemical properties using CV revealed the high electrical conductivity of DGP electrode. The DGP surpasses the graphene prepared with the Hummer method in the morphological and the electrochemical properties that makes it preferable in optoelectronic applications. Moreover, the high quality nanosheets obtained can replace ITO as a highly conductive layer forming a glass electrode for photovoltaic.

## Figures and Tables

**Table 1:** Results of elemental analysis of RGO and DGP samples.

Sample	N %	C %	S %	H %	O %
RGO	2.159	64.59	2.2	6.4	24.6
DGP	0.876	89.99	0.335	1.2	7.6

**Table 2:** Electrical conductivity values for graphite (GPH), graphene oxide (GO), reduced graphene oxide (RGO) and dispersed graphene nanoplatelets (DGP).

Analysis	DGP	RG	GO	GPH
Electrical Conductivity (S/cm)	23.5	0.19	1.9 × 10^−6^	10

## References

[1] Novoselov K.S., Geim A.K., Morozov S.V., Jiang D., Zhang Y., Dubonos S.V., Grigorieva I.V., Firsov A.A. (2004). Electric field effect in atomically thin carbon films. Science.

[2] Balandin A., Ghosh S., Bao W., Calizo I., Teweldebrhan D., Miao F., Lau C.N. (2008). Superior Thermal Conductivity of Single-Layer Graphene. Nano Lett..

[3] Bolotin K., Sikes K.J., Jiang Z., Klima M., Fudenberg G., Hone J., Kim P., Stormer H.L. (2008). Ultrahigh electron mobility in suspended graphene. Solid State Commun..

[4] Lee C., Wei X., Kysar J., Hone J. (2008). Measurement of the elastic properties and intrinsic strength of monolayer graphene. Science.

[5] Stoller M.D., Park S., Zhu Y., An J., Ruoff R.S. (2008). Graphene-Based Ultracapacitors. Nano Lett..

[6] Chae H.K., Siberio-Perez D.Y., Kim J., Go Y., Eddaoudi M., Matzger A.J., O’Keeffe M., Yaghi O.M. (2004). A route to high surface area; porosity and inclusion of large molecules in crystals. Nature.

[7] Kang X., Wang J., Wu H., Liu J., Aksay I.A., Lin Y.A. (2010). A graphene-based electrochemical sensor for sensitive detection of paracetamol. Talanta.

[8] Patchkovskii S., Tse J.S., Yurchenko S.N., Zhechkov L., Heine T., Seifert G. (2005). Graphene nanostructures as tunable storage media for molecular hydrogen. Proc. Natl. Acad. Sci. USA.

[9] Schedin F., Geim A.K., Morozov S.V., Hill E.W., Blake P., Katsnelson M.I., Novoselov K.S. (2007). Detection of individual gas molecules adsorbed on graphene. Nat. Mater..

[10] Dua V., Surwade S.P., Ammu S., Agnihotra S.R., Jain S., Roberts K.E., Park S., Ruoff R.S., Manohar S.K. (2010). All-organic vapor sensor using inkjet-printed reduced graphene oxide. Angew. Chem. Int. Ed..

[11] Avouris P., Chen Z.H., Perebeinos V. (2007). Carbon-based electronics. Nat. Nanotechnol..

[12] Eda G., Chhowalla M. (2009). Graphene-based Composite Thin Films for Electronics. Nano Lett..

[13] Ramanathan T., Abdala A., Stankovich S., Dikin D., Herrera-Alonso M., Piner R.D., Adamson D.H., Schniepp H.C., Chen X., Ruoff R.S. (2008). Functionalized graphene sheets for polymer nanocomposites. Nat. Nanotechnol..

[14] Scheuermann G., Rumi L., Steurer P., Bannwarth W., Mülhaupt R. (2009). Palladium Nanoparticles on Graphite Oxide and Its Functionalized Graphene Derivatives as Highly Active Catalysts for the Suzuki−Miyaura Coupling Reaction. J. Am. Chem. Soc..

[15] Wang X., Zhi L., Müllen K. (2008). Transparent; Conductive Graphene Electrodes for Dye-Sensitized Solar Cells. Nano Lett..

[16] Wu J., Agrawal M., Becerril H.A., Bao Z.N., Liu Z., Chen Y., Peumans P. (2010). Organic Light-Emitting Diodes on Solution-Processed Graphene Transparent Electrodes. ACS Nano.

[17] Echtermeyer T., Britnell L., Jasnos P., Lombardo A., Gorbachev R.V., Grigorenko A.N., Geim A.K., Ferrari A.C., Novoselov K.S. (2011). Strong plasmonic enhancement of photovoltage in graphene. Nat. Commun..

[18] Konstantatos G., Badioli M., Gaudreau L., Osmond J., Bernechea M., de Arquer F.P.G., Gatti F., Koppens F.H.L. (2012). Hybrid graphene-quantum dot phototransistors with ultrahigh gain. Nat. Nanotechnol..

[19] Bae S., Kim H., Lee Y., Xu X., Park J., Zheng Y., Balakrishnan J., Lei T., Ri Kim H., Song Y.I. (2010). Roll-to-roll production of 30-inch graphene films for transparent electrodes. Nat. Nanotechnol..

[20] Kim K., Zhao Y., Jang H., Lee S., Kim J., Kim K.S., Ahn J.-H., Kim P., Choi J.-Y., Hong B.H. (2009). Large-scale pattern growth of graphene films for stretchable transparent electrodes. Nature.

[21] Chen L., Hernandez Y., Feng X., Müllen K. (2012). From nanographene and graphene nanoribbons to graphene sheets: Chemical synthesis. Angew. Chem. Int. Ed..

[22] León V., Quintana M., Herrero M.A., Fierro J., de la Hoz A., Prato M., Vázquez E. (2011). Few-layer graphenes from ball-milling of graphite with melamine. Chem. Commun..

[23] Novoselov K., Jiang D., Schedin F., Booth T., Khotkevich V., Morozov S., Geim A. (2005). Two-dimensional atomic crystals. Proc. Natl. Acad. Sci. USA.

[24] Jiang H. (2011). Chemical preparation of graphene-based nanomaterials and their applications in chemical and biological sensors. Small.

[25] Rao C., Subrahmanyam K.S., Ramakrishna Matte H., Maitra U., Moses K., Govindaraj A. (2011). Graphene: Synthesis; functionalization and properties. Inter. J. Modern Phys. B.

[26] Coleman J. (2013). Liquid Exfoliation of Defect-Free Graphene. Acc. Chem. Res..

[27] Ciesielski A., Samor P. (2014). Graphene via sonication assisted liquid-phase exfoliation. Chem. Soc. Rev..

[28] An X., Simmons T., Shah R., Wolfe C., Lewis K.M., Washington M., Nayak S.K., Talapatra S., Kar S. (2010). Stable aqueous dispersions of noncovalently functionalized graphene from graphite and their multifunctional high-performance applications. Nano Lett..

[29] Sahoo S., Hatui G., Bhattacharya P., Dhibar S., Das C.K. (2013). One Pot Synthesis of Graphene by Exfoliation of Graphite in ODCB. Graphene.

[30] Yang W., Widenkvist E., Jansson U., Grennber H. (2011). Stirring-induced aggregation of graphene in suspension. New J. Chem..

[31] Bourlinos A., Georgakilas V., Zboril R., Steriotis T.A., Stubos A.K. (2009). Liquid-phase exfoliation of graphite towards solubilized graphenes. Small.

[32] Hamilton C.E., Lomeda J.R., Sun Z.Z., Tour J., Barron A. (2009). High-yield organic dispersions of unfunctionalized graphene. Nano Lett..

[33] Zhang X., Coleman A., Katsoni N., Browne W., van Wees B., Feringa B. (2010). Dispersion of graphene in ethanol using a simple solvent exchange method. Chem. Commun..

[34] Nuvoli D., Valentini L., Alzari V., Scognamillo S., Bon S., Piccinini M., Illescas J., Mariani A. (2011). High concentration few layer graphene sheets obtained by liquid phase exfoliation of graphite in ionic liquid. J. Mater. Chem..

[35] O’Neill A., Khan U., Nirmalraj P., Boland J., Coleman J. (2011). Graphene Dispersion and Exfoliation in Low Boiling Point Solvents. J. Phys. Chem. C.

[36] Israelachvili J. (2011). Intermolecular and Surface Forces.

[37] Lotya M., King P., Khan U., De S., Coleman J. (2010). High-Concentration; Surfactant-Stabilized Graphene Dispersions. ACS Nano.

[38] Skaltsas T., Karousis N., Yan H., Wang C., Pispas S., Tagmatarchis N. (2012). Graphene exfoliation in organic solvents and switching solubility in aqueous media with the aid of amphiphilic block copolymers. J. Mater. Chem..

[39] Zhang H., Wen J., Meng X., Yao Y., Yin G.F., Liao X., Huang Z.B. (2012). An Improved Method to Increase the Concentration of Graphene in Organic Solvent. Chem. Lett..

[40] Noroozi M., Zakaria A., Radiman S., Abdul Waha Z. (2016). Environmental Synthesis of Few Layers Graphene Sheets Using Ultrasonic Exfoliation with Enhanced Electrical and Thermal Properties. PLoS ONE.

[41] Takayanagi T., Morimoto M., Yabutani T. (2013). Micellar electrokinetic chromatography of graphene and chemically modified graphenes with dodecylbenzenesulfonate. Anal. Sci..

[42] Song Y., Lee H., Ko J., Ryu J., Kim M., Sohn D. (2014). Preparation and Characterization of Surfactant-Exfoliated Graphene. Bull. Korean Chem. Soc..

[43] Lotya M., Hernandez Y., King P., Smith R., Nicolosi V., Karlsson L., Blighe F.M., De S., Wang Z., McGovern I.T. (2009). Liquid phase production of graphene by exfoliation of graphite in surfactant/water solutions. J. Am. Chem. Soc..

[44] Sukumaran S., Jinesh K., Gopchandran K. (2017). Liquid phase exfoliated graphene for electronic applications. Mater. Res. Express.

[45] Yoon G., Seo D., Ku K., Kim J., Jeon S., Kang K. (2015). Factors Affecting the Exfoliation of Graphite Intercalation Compounds for Graphene Synthesis. Chem. Mater..

[46] Schmaltz B., Weil T., Müllen K. (2009). Polyphenylene-based materials: Control of the electronic function by molecular and supramolecular complexity. Adv. Mater..

[47] De S., King P., Lotya M., O’Neill A., Doherty E., Hernandez Y., Duesberg G., Coleman J. (2010). Flexible; transparent; conducting films of randomly stacked graphene from surfactant stabilized; oxide-free graphene dispersions. Small.

[48] Yeon C., Yun S., Lee K., Lim J. (2015). High-yield graphene exfoliation using sodium dodecyl sulfate accompanied by alcohols as surface-tension-reducing agents in aqueous solution. Carbon.

[49] Bourlinos A., Georgakilas V., Zboril R., Steriotis T., Stubos A., Trapalis C. (2009). Organic functionalisation of graphenes. Solid State Commun..

[50] Guardia L., Fernandez-Merino M., Paredes J., Solis-Fernandez P., Villar-Rodil S., Martinez-Alonso A., Tascon J. (2011). High-throughput production of pristine graphene in an aqueous dispersion assisted by non-ionic surfactants. Carbon.

[51] Liang Y., Hersam M. (2010). Highly Concentrated Graphene Solutions via Polymer Enhanced Solvent Exfoliation and Iterative Solvent Exchange. J. Am. Chem. Soc..

[52] Lockwood N., de Pablo J., Abbott N. (2005). Influence of Surfactant Tail Branching and Organization on the Orientation of Liquid Crystals at Aqueous−Liquid Crystal Interfaces. Langmuir.

[53] Nicolosi V., Chhowalla M., Kanatzidis M., Strano M., Coleman J. (2013). Liquid exfoliation of layered materials. Science.

[54] Jung I., Dikin D., Piner R., Ruoff R. (2008). Tunable electrical conductivity of individual graphene oxide sheets reduced at “low” temperatures. Nano Lett..

[55] Park S., An J., Jung I., Piner R., An S., Li X., Velamakanni A., Ruoff R. (2009). Colloidal suspensions of highly reduced graphene oxide in a wide variety of organic solvents. Nano Lett..

[56] Mativetsky J., Liscio A., Treossi E., Orgiu E., Zanelli A., Samor P., Palermo V. (2011). Graphene Transistors via in Situ Voltage-Induced Reduction of Graphene-Oxide under Ambient Conditions. J. Am. Chem. Soc..

[57] Tölle F., Fabritius M., Mülhaupt R. (2012). Emulsifier-free graphene dispersions with high graphene content for printed electronics and freestanding graphene films. Adv. Funct. Mater..

[58] Stankovich S., Dikin D., Piner R., Kohlhaas K., Kleinhammes A., Jia Y., Wu Y., Nguyen S.T., Ruoff R.S. (2007). Synthesis of graphene-based nanosheets via chemical reduction of exfoliated graphite oxide. Carbon.

[59] Zalan Z., Lazar L., Fueloep F. (2005). Chemistry of hydrazinoalcohols and their heterocyclic derivatives. Part 1. Synthesis of hydrazinoalcohols. Curr. Org. Chem..

[60] Schniepp H., Li J., McAllister M., Sai H., Herrera-Alonso M., Adamson D., Prud’homme R.K., Car R., Saville D.A., Aksay I.A. (2006). Functionalized single graphene sheets derived from splitting graphite oxide. J. Phys. Chem. B.

[61] Mattevi C., Eda G., Agnoli S., Miller S., Mkhoyan K., Celik O., Mastrogiovanni D., Granozzi G., Garfunkel E., Chhowalla M. (2009). Evolution of electrical; chemical; and structural properties of transparent and conducting chemically derived graphene thin films. Adv. Funct. Mater..

[62] Frank O., Mohr M., Maultzsch J., Thomsen C., Riaz I., Jalil R., Novoselov K.S., Tsoukleri G., Parthenios J., Papagelis K. (2011). Raman 2D-Band Splitting in Graphene: Theory and Experiment. ACS Nano.

[63] Abdolhosseinzadeh S., Asgharzadeh H., Kim H. (2015). Fast and fully-scalable synthesis of reduced graphene oxide. Sci. Rep..

[64] Graf D., Molitor F., Ensslin K., Stampfer C., Jungen A., Hierold C., Wirtz L. (2007). Spatially Resolved Raman Spectroscopy of Single- and Few-Layer Graphene. Nano Lett..

[65] Dresselhaus M., Jorio A., Hofmann M., Dresselhaus G., Saito R. (2010). Perspectives on carbon nanotubes and graphene Raman spectroscopy. Nano Lett..

[66] Nawaz K., Ayub M., Khan M., Hussain A., Malik A., Niazi M., Hussain M., Khan A., Ul-Haq N. (2016). Effect of Concentration of Surfactant on the Exfoliation of Graphite to Graphene in Aqueous Media. Nanomater. Nanotechnol..

[67] Khan U., O’Neill A., Lotya M., De S., Coleman J. (2010). High-Concentration Solvent Exfoliation of Graphene. Small.

[68] Li D., Muller M., Gilje S., Kaner R., Wallace G. (2008). Processable aqueous dispersions of graphene nanosheets. Nat. Nanotechnol..

[69] Viana R., da Silva A., Pimentel A. (2012). Infrared Spectroscopy of Anionic; Cationic; and Zwitterionic Surfactants. Adv. Phys. Chem..

[70] Paredes J., Villar-Rodil S., Martinez-Alonso A., Tascon J. (2008). Graphene Oxide Dispersions in Organic Solvents. Langmuir.

[71] Kashyap S., Mishra S., Behera S.K. (2014). Aqueous Colloidal Stability of Graphene Oxide and Chemically Converted Graphene. J. Nanopart..

[72] Abdelsayed V., Moussa S., Hassan H., Aluri H., Collinson M., Samy E.M. (2010). Photothermal Deoxygenation of Graphite Oxide with Laser Excitation in Solution and Graphene-Aided Increase in Water Temperature. J. Phys. Chem. Lett..

